# PAH Exposure

**DOI:** 10.1289/ehp.0800445

**Published:** 2009-04

**Authors:** Paul A. Fowler, Peter J. O’Shaughnessy, Stewart M. Rhind, Jon Ayres

**Affiliations:** Centre for Reproductive Biology & Medicine Institute for Medical Sciences, University of Aberdeen, Aberdeen, United Kingdom, E-mail: p.a.fowler@abdn.ac.uk; Institute of Comparative Medicine, University of Glasgow Veterinary School, Glasgow, United Kingdom, E-mail: p.j.oshaughnessy@vet.gla.ac.uk; Macaulay Institute, Aberdeen, United Kingdom, E-mail: s.rhind@macaulay.ac.uk; Institute of Occupational & Environmental Medicine, University of Birmingham, Birmingham, United Kingdom, E-mail: j.g.ayres@bham.ac.uk

We were very interested to read the article by Choi et al. (2008). The difference between maternal exposure and our own data on actual concentrations of poly cyclic aromatic hydrocarbons (PAHs) in the human male fetal liver (Fowler et al. 2008) was striking. Eight of the PAH exposures meas ured by Choi et al. were also on our list of PAHs measured in the human fetal liver during the second trimester.

Assuming that the 48-hr samples of airborne PAH exposure used by Choi et al. (2008) truly reflect longer-term exposure more relevant to the outcomes under consideration (which is contentious because the measurements may either over estimate or underestimate true exposure), then we can approximate a comparison between the two studies. Therefore, we calculated the fold- difference between the maximal second trimester exposures (nanograms per cubic meter) reported by Choi et al. in their Table 2 and the mean male fetal liver values presented in our Table 3 [(Fowler et al. 2008), corrected to nanograms per kilogram dry weight]. We calculated values separately for fetuses from mothers who smoked cigarettes and for those who did not (Table 1). The smallest difference was 5-fold for benzo[*a*]pyrene (BaP), whereas the largest difference was 8,340-fold for benz[*a*]anthracene (BaA), in all cases representing accumulation in the fetal liver considerably above personal maternal exposure to airborne PAHs. Of course there are other sources of exposure to PAHs, such as air pollution and occupational sources, but these data very clearly suggest that large quantities of PAHs are crossing the placenta and accumulating in the fetus. Perhaps even more interesting was the very different relative proportions of these eight PAHs in the air compared with in fetal livers: BaA comprised 11% in air but 94–96% in the livers, whereas pyrene was 17% in the air but below detection in the livers. This suggests that very different proportions of PAHs are accumulating in fetal tissues and it also underscores the fundamental principle that to really understand health risks we cannot afford to ignore the actual tissue levels in favor of exposure estimates alone.

## Figures and Tables

**Table 1:** Maximum air PAH exposure (ng/m^3^) compared with mean human male fetal liver PAH levels (ng/kg dry weight) from mothers who did and did not smoke, during the second trimester.

	PAH concentration			Relative proportion of PAH		
PAH	Maximum air (ng/m^3^)	Liver mean		Maximum air (ng/m^3^)	Liver mean	
		Nonsmoker	Smoker		Nonsmoker	Smoker
BaA	39.69	331,000	195,000	11.14	95.47	93.80
Benzo[*b*]fluoranthene	67.47	1,300	1,800	18.93	0.38	0.87
Benzo[*k*]fluoranthene	20.33	500	1,000	5.71	0.14	0.48
Benzo[*ghi*]perylene	32.26	800	200	9.14	0.23	0.10
BaP	42.23	500	200	11.85	0.14	0.09
Chrysene	31.13	11,000	9,000	8.73	3.17	4.33
Dibenzo[*a,h*]anthracene	10.76	800	200	3.02	0.24	0.09
Indeno[1,2,3-*cd*]pyrene	50.20	800	500	14.09	0.23	0.24
Pyrene	61.96	0^a^	0	17.39	0	0

a Below detection in human fetal livers.
